# Kaszel Przewlekły u Dzieci

**DOI:** 10.34763/devperiodmed.20182204.329340

**Published:** 2019-01-14

**Authors:** Katarzyna Ptak, Ewa Cichocka-Jarosz, Przemko Kwinta

**Affiliations:** 1 Klinika Chorób Dzieci, Uniwersytecki Szpital Dziecięcy w Krakowie, Polska; 2 Klinika Chorób Dzieci Katedry Pediatrii, Uniwersytet Jagielloński Collegium Medicum, Krakowie Polska

**Keywords:** kaszel przewlekły, kaszel swoisty, kaszel nieswoisty, cough hypesensivity syndrom, monitorowanie kaszlu, chronic cough, specific cough, nonspecific cough, cough hypersensitivity syndrome, monitoring cough

## Abstract

*Kaszel stanowi jedną z najczęstszych dolegliwości zgłaszanych przez pacjentów w gabinecie lekarskim. W ciągu roku większość dzieci może doświadczać 5-8 epizodów kaszlu trwających około tygodnia. Dłuższy niż 4 tygodnie czas trwania kaszlu, definiowany jako kaszel przewlekły, istotnie niepokoi rodziców, pogarsza jakość życia rodziny i stanowi przyczynę zwiększenia częstości konsultacji lekarskich oraz zdarzeń niepożądanych z powodu nieprawidłowego stosowania leków. Stanowi więc istotny problem nie tylko zdrowotny, ale też społeczny*.

*W poniższej pracy został przedstawiony aktualny stan wiedzy dotyczący patofizjologii kaszlu przewlekłego, najnowszych możliwości diagnostycznych, sposobów monitorowania kaszlu i zaleceń postępowania terapeutycznego. Dla zaakcentowania odrębności patomechanizmów prowadzących do stanu kaszlu przewlekłego użyto nowego pojęcia cough hipersensitivity syndrom. Podkreślono fakt, że poszczególne metody monitorowania nasilenia kaszlu nie pozwalają na pełną charakterystykę kaszlu. Aktualnie zaleca się stosowanie jednocześnie kilku metod celem dokonania pełnej oceny. Przedstawiono nowe strategie terapeutyczne opierające się na regulacji przewodzenia bodźców drogą aferentną nerwu błędnego oraz na modyfikowaniu aktywności neurotransmiterów w pniu mózgu i śródmózgowiu. Ponadto w poniższej pracy przedstawiono algorytm postępowania diagnostyczno-terapeutycznego w przypadku kaszlu przewlekłego u dzieci, co pozwala przyspieszyć ustalenie właściwego rozpoznania, rozpoczęcie leczenia oraz poprawić jakość życia pacjentów z problemem kaszlu przewlekłego*.

## Abstract (en)

*Coughing is one of the most common patient complaints at physicians’ office. The majority of children experience 5 to 8 episodes of cough lasting about a week throughout the year. Episodes of cough which last longer than 4 weeks, defined as a chronic cough, result in serious parental concern, impaired quality of life, increased number of medical consultations and the adverse effects of inappropriately used medications. Overall, a chronic cough is not only a serious health problem, but also a social one*.

*The article presented below summarizes our current knowledge on the pathophysiology of chronic cough, the latest diagnostics and most recent measurement and monitoring methods as well as recommendations for therapeutic proceedings. In order to emphasize the distinct pathophysiology of chronic cough we use a new term: cough hypersensitivity syndrome. We point out the necessity of the concurrent implementation of more than one cough monitoring method for its more adequate evaluation. This article in addition presents the diagnostic and therapeutic algorithms in the treatment of a chronic cough which shorten the time to make a proper diagnosis, enable the introduction of adequate treatment, and ultimately improve the patients’ quality of life. We present new therapeutic strategies, which are based on regulating the activity of vagal afferent nerves and modifying the neurotransmiters’ transmission in the brainstem and midbrain*.

## Wprowadzenie

Kaszel jest jednym z najistotniejszych odruchów obronnych, a zaburzenie tego odruchu odgrywa istotną rolę w patogenezie chorób układu oddechowego. Kaszel jest objawem niespecyficznym i może być wywołany wieloma czynnikami, najczęściej jednak w populacji dzieci są to czynniki infekcyjne. Uważa się że w ciągu roku większość dzieci może doświadczać 5-8 epizodów kaszlu trwających około tygodnia. Kaszel jest więc jedną z najczęstszych przyczyn konsultacji w gabinecie pediatrycznym [1], istotnie podwyższa częstość konsultacji lekarskich i zdarzeń niepożądanych z powodu nieprawidłowego stosowania leków [2], a z uwagi na duży wpływ na pogorszenie jakości życia zarówno pacjenta jak i jego rodziny stanowi istotny problem społeczny [3].

## Kaszel definicja i podział

Zgodnie z definicją, kaszel jest odruchem obronnym służącym oczyszczaniu dróg oddechowych z nadmiaru wydzieliny, cząstek pyłów czy innych ciał obcych. Może być też wywołany świadomie. Przyjęto następujące kryteria podziału:

1. Czas trwania: Według *British Thoracic Society* podobnie jak *National Institute for Health and Care Excellence (NICE)* wyróżnia się a) kaszel ostry trwający 3 tygodnie, b) podostry 3-8 tygodni oraz c) przewlekły utrzymujący się ponad 8 tygodni. Według *American College of Chest Physicians* proponowany jest podział na ostry trwający do 4 tygodni i przewlekły utrzymujący się dłużej [4].
2. Charakter: nieproduktywny (suchy) w którym brak odkrztuszania plwociny oraz produktywny (wilgotny) z obecnością plwociny [5].
3. Etiologię: swoisty czyli charakterystyczny dla danej jednostki chorobowej, wymagający leczenia przyczynowego oraz nieswoisty o niezidentyfikowanej przyczynie często ustępujący samoistnie [4].

## Epidemiologia

Pomimo powszechnego występowania kaszlu, badania epidemiologiczne dotyczące kaszlu przewlekłego w populacji dzieci są nieliczne, a ich wyniki różnią się wzajemnie. Jest to konsekwencja korzystania z rożnych klasyfikacji kaszlu, braku powszechnego stosowania obiektywnych metod oceny kaszlu oraz jego dużej zmienności populacyjnej. Na podstawie piśmiennictwa ogólna częstość kaszlu w populacji dzieci waha się od 1-28% [6, 7]. Wraz z wiekiem wzrasta częstość kaszlu niezwiązanego z infekcją (z 34% do 55%), wywołanego wysiłkiem fizycznym (z 10% do 26%), narażeniem na roztocze kurzu domowego (z 2% do 14%) czy zanieczyszczenie powietrza (z 10% do 15%) [8]. Kaszel występuje także obiektywnie częściej u dzieci z *wheezingiem* [8], dzieci rodziców palących tytoń [9] oraz dzieci mieszkających w zanieczyszczonym środowisku lub w warunkach o niskim statusie socjoekonomicznym [10].

## Patofizjologia

Pod względem patofizjologii kaszel jest łukiem odruchowym powstającym w wyniku pobudzenia receptorów będących czuciowymi zakończeniami nerwu błędnego, zlokalizowanymi w górnych i dolnych drogach oddechowych z pominięciem pęcherzyków płucnych. Receptory kaszlu obecne są również w osierdziu, przełyku, przeponie, a także w przewodzie słuchowym zewnętrznym [5] (rycina 1). Wyróżniono 4 rodzaje receptorów kaszlu w drogach oddechowych. Dwa z nich stanowią początek włókien zmielinizowanych A delta i są mechanoreceptorami szybko adaptującymi się (*rapidly adapting receptors, RAR*) oraz wolno adaptującymi się (*slowly adapting receptors* SAR). Kolejne dwa to receptory (TRP, PR)włókien niezmielinizowanych C oskrzeli i tkanki płucnej (tabela I). Główną rolę w inicjacji kaszlu odgrywają receptory szybko adaptujące się (RAR), zlokalizowane podnabłon-kowo w tchawicy i głównych oskrzelach, oraz receptory kanałowe i purynergiczne włókien C: TRPV1 (*transient receptor potentian vanilloid-1*), TRPA1 (*transient receptor potential ankyrin-1*) oraz P2Y i P2X (*purynergic receptor*) rozsiane w tchawicy, drzewie oskrzelowym, a także w wielu innych tkankach organizmu. Receptory RAR pobudzane są mechanicznie, nagłym i znacznym rozciąganiem płuc, a także pośrednio przez czynniki chemiczne tj. dym tytoniowy czy pyły zanieczyszczające atmosferę. W przeciwieństwie do receptorów RAR, receptory włókien C są mało wrażliwe na bodźce mechaniczne, a ich główna rola polega na odbieraniu bodźców chemicznych [4, 5]. TRPV1 są szczególnie wrażliwe na działanie wybranych substancji np. kapsaicyny, TRPA1 na składniki dymu tytoniowego, zanieczyszczenia powietrza, czy temperaturę powietrza poniżej 17 stopni. Na poziomie komórkowym receptory P2Y i P2X reagują na wzrost stężenia ATP, do którego dochodzi w przebiegu uszkodzenia komórki [11]. Działanie receptorów włókiem C związane jest z działaniem pompy Na-K zależnej od ATP, ulegającej otwarciu w odpowiedzi na szereg bodźców powodujących zmiany potencjału błonowego [4].

**Ryc. 1:**
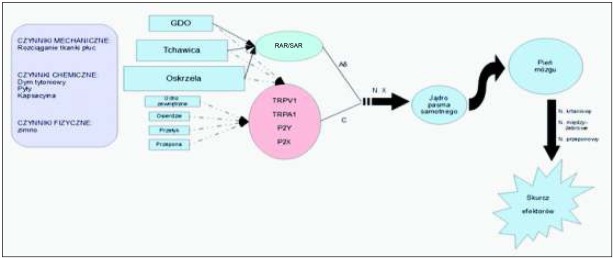
Odruch kaszlu Fig. 1. Cough reflux pathway

**Table I:** Characteristic of receptors in cough reflex pathway [4, 5] Tabela I. Charakterystyka receptorów biorących udział w odruchu kaszlowym [4, 5]

Receptor *Receptors*	Rodzaj receptora *Type of receptors*	Rodzaj włókna nerwowego *Type of nerve fibre*	Prędkość przewodzenia *Conduction velocity*	Czynnik pobudzający *Stimulus*
**RAR^1^**	Mechanoreceptor *Mechanoreceptors*	A delta zmielinizowane *A delta myelinated*	Szybkoprzewodzące (2-30 m/s) Szybkoadaptujące *Fast (2-30 m/s)* *Rapidly adapting*	Zmiana objętości płuc Skurcz oskrzeli Zwiększenie wydzielania śluzu *Respons to: Lung deflection* Bronchoconstriction *Mucose overproduction*
**SAR^2^**	Mechanoreceptor *Mechanoreceptor*	A delta zmielinizowane *A delta myelinated*	Szybkoprzewodzące (2-30 m/s) Wolnoadaptujące *Fast (2-30 m/s)* *Slowly adapting*	Odruch Breuer-Hernig-pobudzenie rec SAR powoduje zahamowanie napędu oddechowego *Involved in: Hering- Breuer’s reflex* - stimulation of SAR receptors terminates inspiration
**TRP^3^*(TRPV1, TRPA1)*** **PR^4^*(P2Y, P2X)***	Chemoreceptor *Chemoreceptor*	C niezmielinizowane *C unmyelinated*	Wolnoprzewodzące (<2 m/s) *Slow (< 2 m/s)*	Bradykinina, kapsacyina, Hipertoniczny r-r soli, Dwutlenek siarki *Bradykinin, capsacicin, Hipertonic saline, Sulphur dioxide*

W niektórych sytuacjach czynniki zewnętrzne jak wirusy, alergeny, substancje drażniące lub inne niespecyficzne czynniki (tj. substancje lotne, zimne powietrze, hyperwentylacja) mogą powodować fenotypowe zmiany w neuronach czuciowych prowadząc do nadwrażliwości na bodźce, które w normalnych warunkach nie wywołują odruchu kaszlu [4, 12]. Prowadzi to do stałego pobudzenia kaszlowego i stanu kaszlu przewlekłego. W modelu zwierzęcym zaobserwowano, że zakażenie wirusem paragrypy typu 3 powoduje znaczący wzrost receptorów TRPV1 zlokalizowanych na włóknach C, co prowadzi do zwiększenia produkcji neuropeptydów jak substancja P czy CGRP (*calcitonin gene-related peptide*) [11]. U pacjentów z kaszlem przewlekłym, bez względu na przyczynę kaszlu, stwierdzono znaczący wzrost stężenia mediatorów zapalnych (histamina, leukotrieny, prostaglandyna E2 i D2) w popłuczynach oskrzelowo-płucnych będący odzwierciedleniem przewlekłego procesu zapalnego [13]. Ponadto długotrwały proces zapalny modyfikuje reakcje immunologiczne prowadząc do rozwoju zapalenia neurogennego. Stan kaszlu przewlekłego zmienia także centralną regulację odruchu kaszlu prowadząc do znaczącego spadku aktywności obszarów mózgu (zakręt obręczy) odpowiedzialnych za hamowanie stymulacji odruchu. Złożony patomechanizm kaszlu przewlekłego doprowadził do wprowadzenie pojęcia *cough hyper-sensivity syndrom* (CHS) dla podkreślenia odrębności procesów leżących u jego podłoża [4, 12].

Bez względu na rodzaj pobudzonego receptora łuk odruchowy kaszlu obejmuje przewodzenie pobudze-nia włóknami aferentnymi nerwu błędnego przez zwój czuciowy górny do części grzbietowo-przyśrodkowej jądra pasma samotnego (pierwsza synapsa odruchu), a następnie do pnia mózgu. Drogę eferentną tworzą włókna ruchowe nerwu błędnego (nerwy krtaniowe) i nerwów rdzeniowych (nerwy międzyżebrowe i nerw przeponowy). Łuk odruchowy kończy się skurczem efektorów – mięśni międzyżebrowych, mięśnia przepony i mięśni krtani [4, 12, 14] (rycina 1). Klinicznie pierwszą przygotowawczą fazą odruchu jest głęboki wdech z towarzyszącym zwiększeniem ciśnienia w jamie brzusznej wskutek skurczu i obniżenia przepony. Następnie dochodzi do gwałtownego wydechu przy zamkniętej głośni, co wiąże się ze wzrostem ciśnienia w klatce piersiowej. Na szczycie wysokiego ciśnienia następuje otwarcie głośni, uniesienie podniebienia miękkiego oraz skurcz mięśni brzucha, co powoduje uniesienie do góry przepony i wyrzut powietrza pod dużym ciśnieniem przez górne drogi oddechowe [4, 12].

### Przyczyny kaszlu przewlekłego

Przyczyny przewlekłego kaszlu są zależne od wieku pacjenta (tabela II). W najmłodszych grupach wiekowych -u noworodków i młodych niemowląt w pierwszej kolejności należy wykluczyć wady rozwojowe układu oddechowego. W okresie małego dziecka do najistotniejszych przyczyn kaszlu zalicza się poinfekcyjną nadreaktywność oskrzeli, a w dalszej kolejności astmę, przewlekające się bakteryjne zapalenie oskrzeli, rozstrzenie oskrzeli, wady wrodzone układu oddechowego. W tej grupie wiekowej należy zawsze wziąć pod uwagę aspirację ciała obcego. Przyczyny kaszlu u nastolatków i dorosłych są zbliżone i najczęściej związane z astmą, zespołem kaszlu z górnych dróg oddechowych, refluksem żołądkowo-przełykowym [6, 15, 16].

Z klinicznego punktu widzenia najistotniejsze znaczenie ma rozróżnienie kaszlu swoistego od nieswoistego. Przyspiesza to postawienie prawidłowego rozpoznania, rozpoczęcie celowanego leczenia oraz uniknięcie, niekiedy nieodwracalnych, zmian w układzie oddechowym. Kaszel swoisty jest objawem typowym dla określonej jednostki chorobowej, a więc jego charakter oraz towarzyszące objawy będą stanowiły podstawę rozpoznania. Dla przyspieszenia wdrożenia prawidłowego postępowania wyróżniono swoiste objawy w badaniu podmiotowym i przedmiotowym tzw. *red flags*, kierujące uwagę lekarza na właściwe rozpoznanie [4, 5] (tabela III). Izolowany kaszel nieswoisty może natomiast być związany z nadreaktywnością receptorów kaszlowych i często ma tendencję do samoistnego ustępowania. Nie wymaga więc specyficznego leczenia. Jednocześnie podkreśla się konieczność stałej kontroli pacjentów z kaszlem nieswoistym ponieważ istnieją sytuacje kliniczne, w których kaszel ten jest w istocie błędnie zdiagnozowanym kaszlem swoistym (np. w przebiegu zespołu kaszlu z górnych dróg oddechowych, astmy, refluksu żołądkowo-przełykowego).

## Diagnostyka

Podstawę w diagnostyce przyczyny kaszlu przewlekłego stanowi prawidłowo zebrany wywiad i badanie fi'zykalne. W badaniu podmiotowym istotne jest ustalenie charakteru kaszlu (produktywny/nieproduktywny), jego specyficznego brzmienia (typu „beczenia kozy” w tracheobronchomegalii lub typu „piania koguta”

**Table II:** Etiologies of chronic cough dependent on age [6,15,16] Tabela II. Przyczyny występowania kaszlu w zależności od wieku [6,15,16]

Wiek	Przyczyna
*Age*	*Etiology*
**Okres noworodkowy *Newborn***	Wady rozwojowe (przetoka tchawiczo-przełykowa,
	pierścień naczyniowy, laryngotracheomalacja, wady oskrzeli i płuc)
	Refluks żołądkowo-przełykowy
	Dysplazja oskrzelowo-płucna
	Narażenie na czynniki drażniące (dym tytoniowy!)
	Zapalenie płuc wywołane *Pneumocistis jiroveci*
	u dzieci immunologicznie niekompetentnych (np. wcześniaki) *Congenital defects (tracheoesophageal fistula, vascular ring*,
	*laryngotracheomalacia, lung and bronchial defects)*
	*Gastroesophageal reflux*
	*Broncho-pulmonary dysplasia*
	*Environmental exposure (tabacco smoke!)*
	*Pneumocistis pneumonia in non immunocompetent children (prematures)*

**Okres niemowlęcy** **Małe dzieci (<4 rż)** ***Infants*** ***Children < 4 years***	Wady rozwojowe (przetoka tchawiczo-przełykowa, wady oskrzeli i płuc)
	Schorzenia neurologiczne z zaburzeniami połykania
	Kaszel poinfekcyjny (CMV, RSV, chlamydia, krztusiec)
	Mukowiscydoza
	Dysplazja oskrzelowo-płucna
	Zachłyśnięcie ciałem obcym
	Refluks żołądkowo-przełykowy
	Astma wczesnodziecięca
	Narażenie na czynniki drażniące (dym tytoniowy)
	Pierwotna dyskineza rzęsek
	*Congenital defects (tracheo-oesophageal fistula, lungs and bronchial defects)*
	*Neurological disorders with dysphagia*
	Post-infectious cough (CMV, RSV, chlamydia, pertusiss)
	Cystic fibrosis
	Broncho-pulmonary dysplasia
	Foreign body aspiration
	Gastroesophageal reflux
	Asthma in young children
	Environmental exposures (tabacco smoke!)
	Primary ciliary dyskinesia

**Wiek przedszkolny** ***Children 4-6 years***	Aspiracja ciała obcego
	Kaszel poinfekcyjny
	Zespół kaszlu z górnych dróg oddechowych
	Astma
	Alergiczny nieżyt nosa
	Mukowiscydoza
	Przewlekające się bakteryjne zapalenie oskrzeli
	Pierwotna dyskineza rzęsek
	Czynniki drażniące (dym tytoniowy!)
	Rozstrzenie oskrzeli
	Wady rozwojowe układu oddechowego
	*Foreign body aspiration*
	*Post-infectious cough*
	*Upper airways cough syndrom*
	*Asthma*
	*Allergic rhinitis*
	*Cystic fibrosis*
	*Protracted bacterial bronchitis*
	*Primary ciliary dyskinesia*
	*Environmental exposures (tabacco smoke!)*
	*Bronchiectasis*
	*Congenital defects*

**Wiek szkolny** ***Children 6-18 years***	Astma
	Zespół kaszlu z górnych dróg oddechowych
	Alergiczny nieżyt nosa
	Refluks żołądkowo-przełykowym
	Zachłyśniecie ciałem obcym
	Kaszel psychogenny
	Czynniki drażniące (dym tytoniowy/palenie tytoniu)
	*Asthma*
	*Upper airways cough syndrom*
	*Allergic rhinitis*
	*Gastroesophageal reflux*
	*Foreign body aspiration*
	*Habit cough*
	*Environmental exposure (tabacco smoke!)*

**Table III:** Indicators of the presence of specific cough [5, 28]. Tabela III. Charakterystyczne cechy sugerujące swoistą przyczynę kaszlu [5, 28].

Dane z wywiadu i/lub badania fizykalnego tzw. „red flags“ *Red flags in history and/or in physical examination*	Prawdopodobne rozpoznanie *Diagnosis*	Wstępne postępowanie diagnostyczne *Preliminary diagnostic tests*
Nagły początek kaszlu,		
• poprzedzony krztuszeniem,		RTG klatki piersiowej
• u dziecka poprzednio zdrowego *Sudden onset*,	Aspiracja ciała obcego *Foreign body aspiration*	Fiberobronchoskopia/Bronchoskopia sztywna
• *Preceeded by episode of choking or prolonged coughing*,		*Chest X-ray Fiberoptic/rigid bronchoscopy*
*• in previously healthy child*		

Kaszel szczekający		
Świst wdechowy		
Bez objawów infekcji	Laryngo/tracheomalacja	Fiberobronchoskopia
Niemowlęta	Pierścień naczyniowy	AngioCT
*Barking cough*	*Laryngo/tracheomalacia*	*Bronchoscopy*
*Stridor*	*Vascular ring*	*Angio CT*
*No infection*		
*Infants*		

Kaszel związany z przyjmowaniem pokarmów u niemowląt *Cough in the course of feeding*	Przetoka tchawiczo-przełykowa *Tracheosophageal fistula*	RTG klatki piersiowej z podaniem kontrastu Bronchofiberoskopia *Chet X-ray with contrast Fiberoptic bronchoscopy*

Kaszel o charakterze „piania koguta“		
Wymioty po kaszlu		
U niemowląt może doprowadzić		
do bezdechu i drgawek	Krztusiec *Pertussis*	Testy serologiczne *Serological tests*
*Whooping cough (inspiratory whoop)*		
*Vomiting after coughing*		
*Apnea or seizures after cough in infants*		
		RTG klatki piersiowej
		RT 23/Test IGRA

Krwioplucie		badanie bakteriologiczne
Utrata masy ciała		plwociny/BAL
Nocne poty	Gruźlica	badanie bakteriologiczne popłuczyn
*Hemoptysis*	*Tuberculosis*	żołądkowych
*Weight loss*		*Chest X-ray*
*Nocturnal sweating*		*TB skin test/TB blood test*
		*Sputum smears and cultures*
		*Gastric washing cultures*

Kaszel produktywny/ suchy		
• po położeniu się i nad ranem		
Blokada nosa		Konsultacja laryngologiczna
Bóle głowy	Przewlekłe zapalenie zatok	TK zatok
*Productive/non- productive cough*	*Chronic rhinosinusitis*	*ENT consultation*
• *worse in morning and when supine*		*Sinus CT*
*Nasal congestion*		
*Headache*		

Kaszel produktywny/ suchy		
• po położeniu się i nad ranem		Konsultacja laryngologiczna
Blokada nosa		
Uczucie spływania wydzieliny	Zespół kaszlu z górnych	Staranna Leki miejscowe toaleta nosa
po tylnej ścianie gardła	dróg oddechowych	Antybiotykoterapia
Tylna ściana gardła granulowana		
*Productive/non- productive cough*	*Post nasal drip syndrome*	*ENT consultation*
• *worse in morning and when supine*	*(PNDS)*	*Proper clearing the mucus*
*Nasal congestion*		*Topical therapy*
*Post nasal drip*		*Antibiotic therapy*
*„Cobblestone” of the pharynx mucosa*		

Kaszel produktywny		
Zgryz otwarty		
Tor oddychania przez otwarte usta		
Blokada nosa		
WZUŚ, deficyt słuchu	Przerost migdałka gardłowego	Fiberoskopia
*Productive cough*	*Adenoid hypertrophy*	*Nasal endoscopy*
*Anterior open bite*		
*Mouth breathing*		
*Nasal congestion*		
*Secretory otitis media, hearing loss*		

Kaszel produktywnym		
Słaby przyrost masy ciała		
Polipy w jamie nosowej (+/-)		HRCT
Palce pałeczkowate (+/-)	Rozstrzenie oskrzeli	FeNO w powietrzu
*Productive cough*	(w tym CF i PCD)	wydychanym z nosa
*Growth retardation*	*Bronchiectases*	*HRCT*
*Nasal polips (+/-)*		*FeNO test*
*Digital clubbing (+/-)*		

Kaszel nieproduktywny		
Świsty wydechowe		Badania czynnościowe
Dobra odpowiedź na beta-2-mimetyki		układu oddechowego
Dodatni wywiad osobniczy/rodzinny	Astma	w tym spirometria z testem
w kierunku atopii		odwracalności obturacji oskrzeli
*Non-productive cough*	*Asthma*	
*Wheezing*		*Spirometry: baseline and*
*Cough resolve after beta-2-agonist therapy*		*bronchodilation test*
*Family and personal history of atopy*		

Tachypnoe		Echokardiogram
Hypoksemia		EKG
Nieprawidłowości w badaniu kardiologicznym	Wada serca	RTG klatki piersiowej
*Tachypnea*	*Congenital heart defect*	*Echocardiography*
*Hypoxemia*		*EC*
*Abnormalities in circulatory system examination*		*Chest X-ray*

Kaszel suchy		
Teatralne pozy		
Występujący tylko w obecności osób trzecich		
Niewystępujący w nocy		
Sytuacje napięcia emocjonalnego w otoczeniu	Kaszel nawykowy	Psychoterapia
*Non-productive cough*	*Psychogenic (Habit) cough*	*Psychotherapy*
*Occure when other people are around*		
*Absent at night*		
*Emotional problems*		

Stosowanie leków	ACEI	Ustąpienie kaszlu po odstawieniu leku
*Pharmacotherapy*	*ACEI*	*Coughing resolved after stopping therapy*

Kaszel produktywny		Morfologia krwi
Nawracające infekcje	Niedobory odporności	Diagnostyka immunologiczna
*Productive cough*	*Immune deficiency*	*Complete blood count*
*Recurrent infections*		*Immunological tests*

w krztuścu), pory doby na którą przypada największe nasilenie kaszlu, sezonowości występowania kaszlu w skali roku. Istotne jest ustalenie objawów towarzyszących jak blokada nosa, krwioplucie, wymioty. U dzieci z istotną klinicznie alergią na alergeny powietrznopochodne ważne jest ustalenie stopnia narażenia na pyłek roślin, pleśnie, roztocze kurzu domowego czy sierść zwierząt. Uzupełnieniem jest wywiad rodzinny w kierunku atopii, narażenie na dym tytoniowy i na zanieczyszczenie powietrza. Badanie fizykalne powinno być ukierunkowane na ewentualnie objawy świadczące o chorobie przewlekłej przebiegającej z kaszlem. Niski wzrost i niedowaga mogą wskazywać na mukowiscydozę czy pierwotne niedobory odporności. Cechy dysmorfii mogą sugerować występowanie wad anatomicznych. W zakresie skóry obecność zmian o typie atopowego zapalenia skóry może świadczyć o atopii i astmie. Mowa nosowa, blokada nosa, tor oddychania przez otwarte usta, granulowanie tylnej ściany gardła (grudki chłonne, spływanie wydzieliny po tylnej ścianie gardła) oraz przerost migdałka gardłowego może korespondować z przewlekłym nieżytem nosa, a obecność polipów nosa sugeruje mukowiscydozę czy wrodzoną dyskinezę rzęsek, podczas gdy wady podniebienia twardego decydują o zwiększonym ryzyku zaburzeń połykania i aspiracji treści pokarmowej do układu oddechowego. Rzadką przyczynę kaszlu przewlekłego może stanowić odruch z kanału słuchowego wywołany nieprawidłowościami dotyczącymi budowy ucha zewnętrznego. W badaniu klatki piersiowej i brzucha należy wykluczyć objawy jak szmer nad sercem, ubytki tętna, hepatomegalię mogące sugerować wadę serca. Dekstrokardia i *situts inversus* zwiększają prawdopodobieństwo rozpoznania zespołu wrodzonej dyskinezy rzęsek.

Szczegółowe badanie klatki piersiowej rozpoczyna się od oglądania. Nieprawidłowości budowy ścian klatki piersiowej jak np. klatka beczkowata występuje w przebiegu niektórych chorób obturacyjnych płuc. Podczas osłuchiwania szczególnie istotna jest ocena symetrii zjawisk opukowych i osłuchowych. Przykładowo jednostronny świst wydechowy lub asymetria osłuchowa mogą sugerować jednostronne zamknięcie drzewa oskrzelowego, np. w przebiegu aspiracji ciała obcego. Polimorficzny *wheezing* z towarzyszącym kaszlem i dobrą odpowiedzią na leki z grupy beta-mimetyków silnie sugeruje astmę, ale może także występować w nadreaktywności oskrzeli, towarzyszyć dysplazji oskrzelowo-płucnej, czy niedoborom odporności, niewydolności serca lub po ostrych zakażeniach układu oddechowego. Monofoniczny świst oddechowy budzi silne podejrzenie obturacji dużych dróg oddechowych wskutek ucisku z zewnątrz np. przez guz śródpiersia czy przez pierścień naczyniowy [4]. Charakterystyczne cechy dla swoistych przyczyn kaszlu podano w tabeli III.

Rodzaj badań dodatkowych uzależniony jest od podejrzenia określonej jednostki chorobowej, jednak u większości pacjentów z kaszlem przewlekłym podstawę diagnostyki stanowi zdjęcie RTG klatki piersiowej i spirometria z testem odwracalności obturacji oskrzeli [17, 18]. Te dwa badania są zazwyczaj łatwo dostępne i często pozwalają ograniczyć dalszą diagnostykę kaszlu wskazując na jego specyficzną przyczynę. Prawidłowy wynik zdjęcia radiologicznego zazwyczaj występuje w przebiegu kaszlu nieswoistego i psychogennego, ale nie wyklucza występowania innych swoistych przyczyn jak astma, aspiracja ciała obcego czy rozstrzenie oskrzeli. Istnieje kilka charakterystycznych objawów radiologicznych, które w zgodności z danymi klinicznymi przemawiają za daną jednostką chorobową. 1) Nadmierne jednostronne upowietrznienie pól płucnych przemawia za obecnością ciała obcego, obustronne za obturacją dolnych dróg oddechowych, w tym za zaostrzeniem astmy. 2) Wzmożony rysunek oskrzelowy z niewielkimi konsolidacjami zapalnymi towarzyszy przewlekającemu się zapaleniu oskrzeli. 3) Niedodma płata środkowego płuca prawego (zespół płata środkowego) występuje w przewlekłych stanach zapalnych, niedoborach odporności, limfadenopatii. 4) Pogrubienie rysunku oskrzeli (objaw „torów tramwajowych”) charakterystyczne jest dla rozstrzeni oskrzeli. 5) Poszerzenie śródpiersia wymaga wzmożonej czujności onkologicznej oraz diagnostyki w kierunku limfadenopatii, poszerzenie sylwetki serca występuje we wrodzonych wadach serca, a poszerzenie tętnic płucnych w pierwotnym lub wtórnym nadciśnieniu płucnym [4].

Badanie spirometryczne z testem odwracalności obturacji, pomimo dużej dostępności jest z reguły możliwe do wykonania u pacjentów powyżej 6. roku życia. Nieprawidłowy wynik badania ma dużą wartość diagnostyczną, ale prawidłowy nie wyklucza patologii. Obturacja dróg oddechowych odwracalna po podaniu beta-2-mimetyku silnie przemawia za astmą, podczas gdy restrykcja za chorobami śródmiąższowymi płuc lub restrykcyjną klatkę piersiową. Badanie powinno być zawsze przeprowadzone najpierw w warunkach podstawowych, następnie po podaniu beta-2-mimetyku, a w przypadku wątpliwości diagnostycznych należy wykonać próbę prowokacji (np. 4,5% NaCl) [20].

Wybór pozostałych testów diagnostycznych uzależniony jest od postawionego wstępnego rozpoznania oraz danych uzyskanych z wywiadu (tabela III). W dalszej kolejności zaleca się wykonanie następujących badań mających na celu określenie swoistej przyczyny kaszlu: morfologia z rozmazem, badania serologiczne (*RSV*, *Mycoplasma pneumoniae, Chlamydophila pneumoniae* i *Bordetella pertusis*), ocena laryngologiczna z ewentualnymi badaniami diagnostycznymi (endoskopia nosogardła, TK zatok), testy skórne z alergenami powietrznopochodnymi, badanie przewodu pokarmowego (pH-metria, impedancja przełyku), konsultacja psychologiczna (w przypadku podejrzenia tła psychogennego) [19, 21]. Dalsza diagnostyka o ile jest wskazana, powinna być poprzedzona konsultacją specjalisty. Zalecane wstępne postępowanie diagnostyczne w swoistych przyczynach kaszlu przedstawia tabela III.

**Ryc. 2:**
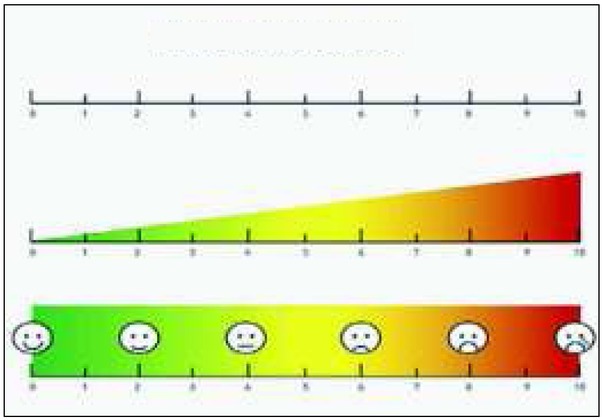
Wizualna skala analogowa: https://tidsskriftet.no/2014/02/sprakspalten/vas-visuell-analog-skala Fig. 2. Visual analogue scale https://tidsskriftet.no/2014/02/sprakspalten/vas-visuell-analog-skala

## Sposoby monitorowania kaszlu

Monitorowanie nasilenia kaszlu służy przede wszystkim ocenie skuteczność zastosowanej terapii, ale także ocenie jakości życia pacjentów z problemem kaszlu przewlekłego. Istnieją wystandaryzowane narzędzia przeznaczone do oceny określonych parametrów kaszlu jak nasilenie, częstotliwość czy wpływ na jakość życia. 1) Do subiektywnych metod oceny nasilenia kaszlu należy a) wizualna skala analogowa (*visual analoque scale − VAS*), w której pacjent zaznacza nasilenie kaszlu na 100 mm skali (lub 10 cm linijce) rozpiętej pomiędzy stanem bez kaszlu, a największym nasileniem kaszlu w życiu. Skala ta jest łatwo dostępna, powtarzalna, a badanie krótkie (rycina 2) [22]. Inną subiektywną metodą jest b) dzienniczek kaszlu (*Cough Diary*), wypełniany codziennie i zawierający pytania dotyczące częstości kaszlu i objawów towarzyszących. Zalecane jest wypełnianie dzienniczka osobiście przez dzieci co zwiększa wiarygodność testu [23]. 2) Do obiektywnych metod oceny częstotliwości kaszlu służą urządzenia automatyczne. Aktualnie na rynku dostępnych jest kilka rodzajów takich urządzeń, jednak tylko dwa z nich mają zastosowanie w klinice [24]. a) *Leicester cough monitor (LCM, Glenfield Hospital, Leicester, UK*) (rycina 3a) składa się z mikrofonu i urządzenia mp3 z możliwością rejestracji dźwięku przez całą dobę. Program automatycznie kwalifikuje nagrane dźwięki jako kaszel.

b) *VitaloJak (Vitalograph Ltd., Buckingham, UK, and University Hospital of South Manchester, UK)* (rycina 3b) składa się z 2 mikrofonów i urządzenia mp3 i również pozwala na całodobową rejestrację, jednak wymaga manualnej kwalifikacji dźwięków jako kaszel [24]. 3) Innym aspektem charakteryzującym kaszel jest wpływ na jakość życia. Służą do tego wystandaryzowane kwestionariusze oceny jakości życia związanej ze stanem zdrowia tzw. *Health-related Quality of life questionnaire* (HRQOL). Do rekomendowanych należą *Leicester cough questionnaire (LCQ)* oraz *cough-specific quality of life questionnaire (CQLQ)* zaprojektowane dla dorosłych [25]. Aktualnie nie istnieje kwestionariusz oceniający wpływ kaszlu na jakość życia w populacji dziecięcej. Dla tej grupy pacjentów zalecane jest użycie ogólnego kwestionariusza *Pediatric Questionnaire (PedQL)* dostępnego odpłatnie w polskiej wersji językowej na stronie http://www.pedsql.org/about_pedsql.html [25]. Złotym standardem w ocenie kaszlu jest stosowanie jednocześnie kilku metod celem dokonania najpełniejszej i najbardziej obiektywnej charakterystyki [25].

## Leczenie

Rozpoznanie swoistego charakteru kaszlu umożliwia rozpoczęcie leczenia celowanego dla danej jednostki chorobowej. Należy podkreślić, że w większości przypadków przewlekłego kaszlu u dzieci ważnym postępowaniem uzupełniającym jest staranna toaleta przewodów nosowych (za pomocą dostępnych aspiratorów lub płukanie nosa). W przypadku kaszlu z górnych dróg oddechowych uzasadnione jest stosowanie leków donosowych. U pacjentów z towarzyszącą obturacją oskrzeli należy ocenić odpowiedź na leczenie beta-mimetykiem [18, 26]. U dzieci z przewlekłym kaszlem produktywnym, którego najczęstszą przyczyną jest przewlekające się bakteryjne zapalenie oskrzeli (PBB), proponuje się włączenie empirycznej antybiotykoterapii; zgodnie z najczęstszą etiologią (*Hemophilus influenzae* i *Streptococcus pneumoniae)* zaleca się stosowanie amoksycyliny z kwasem klawulanowym przez 2 tygodnie, a przy braku poprawy kontynuację terapii przez kolejne 2 tygodnie [19, 26]. U pacjentów, u których pomimo stosowania antybiotyków beta-laktamowych przez 4 tygodnie nie uzyskano ustąpienia objawów można rozważyć zastosowanie azytromycyny [19, 26] Zalecane postępowanie w przypadku produktywnego kaszlu przewlekłego przedstawiono w postaci Algorytmu (rycina 4). W większości przypadków przewlekłego kaszlu nieproduktywnego z uwagi na jego samoograniczający charakter nie jest zalecana antybiotykoterapia. W tej grupie pacjentów można rozważyć próbę diagnostyczno-terapeutyczną z użyciem wziewnych glikokortykosteroidów w średniej dawce stosowanych początkowo przez 2 tygodnie, z oceną odpowiedzi na leczenie. W przypadku nawrotu kaszlu po zakończeniu terapii i wywiadu wskazującego na astmę wczesnodziecięcą, przyjmuje się za uzasadnione wydłużenie stosowania wziewnych glikokortykosteroidów do 3 miesięcy, równolegle z rozpoczęciem diagnostyki astmy i uwzględnieniem diagnostyki różnicowej [26]. Zalecane postępowanie w przypadku nieproduktywnego kaszlu przewlekłego przedstawiono w postaci Algorytmu (rycina 5). Aktualnie nie ma zaleceń co do diagnostyczno-terapeutycznego stosowania leków przeciwrefluksowych, przeciwhistaminowych, wziewnych beta-2-mimetyków czy antagonistów receptorów leukotrienowych. U dzieci nie jest zgodne z rejestracją produktów leczniczych stosowanie opioidowych leków przeciw-kaszlowych tj. morfina czy kodeina, a także nieopioidowych leków tj. dextrometorfan [27]. Jakkolwiek z uwagi na ich działanie neuromodulujące mogą one odgrywać rolę w leczeniu *cough hipersensitivity syndrome* [13].

**Ryc. 3:**
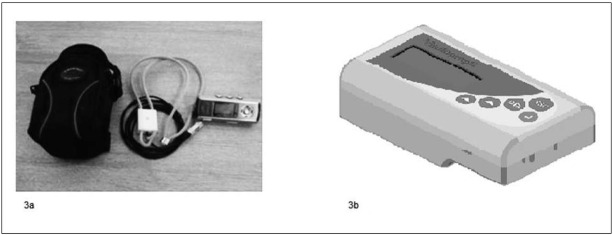
Urządzenia służące monitorowaniu nasilenia kaszlu. (3a) Leicester cough monitor; https://doi.org/10.1155/2018/9845321; (3b) VitaloJak : https://vitalograph.com/news/28/vitalojak-wins-north-west-nhs-innovation-award Fig. 3. Cough monitoring systems: (3a) Leicester cough monitor; https://doi.org/10.1155/2018/9845321; (3b) VitaloJak : https://vitalograph.com/news/28/vitalojak-wins-north-west-nhs-innovation-award

Należy zawsze pamiętać, że u każdego dziecka, a w szczególności u pacjentów z kaszlem przewlekłym, należy wyeliminować narażenie na czynniki drażniące, a w szczególności na dym tytoniowy.

## Nowe strategie terapeutyczne

Aktualnie prowadzonych jest wiele badań klinicznych z zastosowaniem nowych preparatów do leczenia kaszlu przewlekłego. Mechanizm działania tych leków polega głównie na regulowaniu przewodzenia bodźców drogą aferentną nerwu błędnego oraz na modyfikowaniu aktywności neurotransmiterów w pniu mózgu i śródmózgowiu. Najbardziej obiecujące wydają się być badania dotyczące antagonistów receptorów adenozyny P2X i P2Y, z których największe znaczenie ma P2X3 (AF-219), zlokalizowanych na zakończeniach włókien C [12, 13]. Inne badania wskazują na efekt przeciwkaszlowy leków hamujących pompę Na-K, do których należą leki przeciwhistaminowe, inhibitory pompy protonowej czy jony cynku [15]. Nie ma jednak zaleceń co do ich stosowania celem opanowania kaszlu. Prowadzone są także badania nad antagonistami receptorów dla kapsaicyny (*Transient receptor potentian vanilloid-1* TRPV1) oraz receptorów wrażliwych na składniki dymu tytoniowego *(Transient receptor potential ankyrin-1 TRPA1)*, jednak jak dotąd nie wykazano skuteczności tych leków [13,18]. Pojawiają się doniesienia dotyczące profilaktycznego stosowania witaminy D ponieważ receptory dla tej witaminy znajdują się na większości komórek organizmu, w tym na komórkach immunologicznie kompetentnych. Witamina D może odgrywać rolę modulatora w regulacji odruchu kaszlu poprzez udział w aktywacji dojrzewania makrofagów, a poprzez wpływ na produkcję katelicydyny wykazuje działanie przeciwbakteryjne [28]. W prospektywnym badaniu obserwacyjnym przeprowadzonym w latach 2014-2015 na populacji dzieci tureckich ustalono że utrzymanie prawidłowego poziomu witaminy D (>20 ng/ml), a w przypadku jej niedoborów szybkie ich uzupełnienie, zmniejsza ryzyko nawracania infekcji dróg oddechowych i kaszlu przewlekłego [28].

**Ryc. 4:**
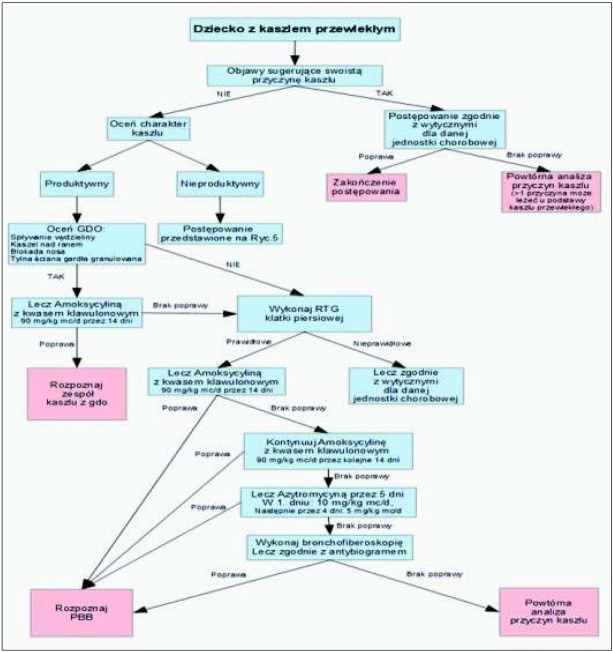
Algorytm postępowania w kaszlu przewlekłym u dzieci. Modyfikacja własna na podstawie Chang et al. *Fig. 4. Suggested algorithm for investigation and management of children with chronic cough. Modified from Chnag et al*.

## Postępowanie

W podejmowaniu decyzji diagnostyczno-terapeutycznych u dzieci z kaszlem przewlekłym zaleca się stosowanie algorytmów pediatrycznych [29,30]. Są one pomocne w szybszym ustaleniu prawidłowego rozpoznania i przyczyniają się do wcześniejszego włączenia właściwego leczenia i poprawy jakości życia [2, 4]. Wyróżniono tzw. „czerwone flagi” („red flags”) celem łatwiejszej identyfikacji pacjentów wymagających pilnego leczenia. Najczęściej używanym jest algorytm zaproponowany przez *American College of Chest Physicians* na podstawie A. Chang *et al*. [30]. W oparciu o ten algorytm poniżej przedstawiamy schemat stosowany w naszym ośrodku (ryciny 4 i 5).

**Ryc. 5:**
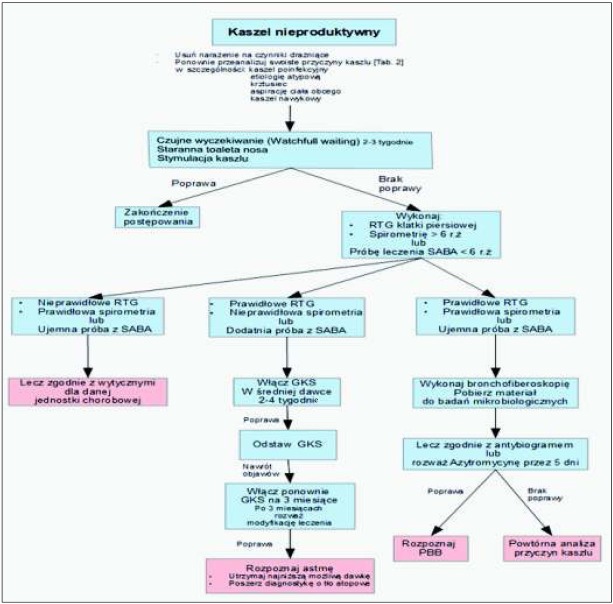
Algorytm postępowania w przewlekłym kaszlu nieproduktywnym u dzieci. Modyfikacja własna na podstawie Chang et al. *Fig. 5. Suggested algorithm for investigation and management of children with non-specific chronić cough. Modified from Chang et al*.
